# Nephrocalcinosis and kidney function in children and adults with X-linked hypophosphatemia: baseline results from a large longitudinal study

**DOI:** 10.1093/jbmr/zjae127

**Published:** 2024-08-16

**Authors:** Anthony A Portale, Leanne Ward, Kathryn Dahir, Pablo Florenzano, Steven W Ing, Suzanne M Jan de Beur, Regina M Martin, Adriana I Meza-Martinez, Neil Paloian, Ambika Ashraf, Bradley P Dixon, Aliya Khan, Craig Langman, Angel Chen, Christine Wang, Mary Scott Roberts, P K Tandon, Camille Bedrosian, Erik A Imel

**Affiliations:** Division of Pediatric Nephrology, University of California San Francisco Benioff Children’s Hospital, San Francisco, CA 94158, United States; Department of Pediatrics, University of Ottawa, Children's Hospital of Eastern Ontario, Ottawa, Ontario, ON K1H 8L1, Canada; Vanderbilt University Medical Center, Nashville, TN 37232, United States; Department of Endocrinology, Faculty of Medicine, Pontificia Universidad Católica de Chile and Centro UC Traslacional en Endocrinología, CETREN-UC, 8320165 Santiago, Chile; Division of Endocrinology, Diabetes, and Metabolism, Ohio State University Wexner Medical Center, Columbus, OH 43210, United States; Division of Endocrinology and Metabolism, Department of Medicine, University of Virginia School of Medicine, Charlotteville, VA 22903, United States; Hospital das Clínicas da Faculdade de Medicina da Universidade de São Paulo, Sao Paulo 05403-010, Brazil; Hospital Infantil Universitario de San José, 111221 Bogota DC, Colombia; University of Wisconsin School of Medicine and Public Health, Madison, WI 53726, United States; University of Alabama at Birmingham, Birmingham, AL 35294, United States; Renal Section, Department of Pediatrics, University of Colorado School of Medicine, Aurora, CO 80045, United States; McMaster University, Hamilton, Ontario L8S 4L8, Canada; Emeritus Professor of Pediatrics, Northwestern Feinberg School of Medicine, Chicago, IL 60611, United States; Ultragenyx Pharmaceutical Inc., Novato, CA 94949, United States; Ultragenyx Pharmaceutical Inc., Novato, CA 94949, United States; Ultragenyx Pharmaceutical Inc., Novato, CA 94949, United States; Ultragenyx Pharmaceutical Inc., Novato, CA 94949, United States; Ultragenyx Pharmaceutical Inc., Novato, CA 94949, United States; Indiana University School of Medicine, Indianapolis, IN 46202, United States

**Keywords:** X-linked hypophosphatemia, renal function, active vitamin D, phosphate, nephrocalcinosis, hyperparathyroidism

## Abstract

**Background:**

In patients with X-linked hypophosphatemia (XLH), conventional therapy with oral phosphate salts and active vitamin D has been associated with nephrocalcinosis. However, the nature of the relationships among XLH, its treatment, nephrocalcinosis, and kidney function remain poorly understood.

**Methods:**

Renal ultrasounds were performed and glomerular filtration rates were estimated (eGFR) at baseline in burosumab-naïve patients with XLH who participated in burosumab clinical trials (NCT02181764, NCT02526160, NCT02537431, NCT02163577, NCT02750618, NCT02915705) or enrolled in the XLH Disease Monitoring Program (XLH-DMP; NCT03651505). In this cross-sectional analysis, patient, disease, and treatment characteristics were described among patients with and without nephrocalcinosis.

**Results:**

The analysis included 196 children (mean [SD] age 7.6 [4.0] yr) and 318 adults (40.3 [13.1] yr). Mean (SD) height z-score was −1.9 (1.2) for children and −2.3 (1.7) for adults. Nearly all children (97%) and adults (94%) had previously received conventional therapy. Nephrocalcinosis was detected in 22% of children and 38% of adults. In children, reduced eGFR <90 mL/min/1.73 m^2^ was more prevalent in those with nephrocalcinosis (25%) than in those without (11%), a finding that was not observed in adults. Children with nephrocalcinosis had lower mean values of TmP/GFR (*p*<.05), serum 1,25(OH)_2_D (*p*<.05), and eGFR (*p*<.001) and higher mean serum calcium concentrations (*p*<.05) than did those without nephrocalcinosis. Adults with nephrocalcinosis had lower mean serum phosphorus (*p*<.01) and 1,25(OH)_2_D (*p*<.05) concentrations than those without. Exploratory logistic regression analyses revealed no significant associations between the presence of nephrocalcinosis and other described patient or disease characteristics.

**Conclusions:**

Nephrocalcinosis was observed in nearly one-quarter of children and more than one-third of adults with XLH. Further study is needed to better understand the predictors and long-term consequences of nephrocalcinosis, with surveillance for nephrocalcinosis remaining important in the management of XLH.

## Introduction

X-linked hypophosphatemia (XLH) is a rare disorder caused by pathogenic variants in the phosphate-regulating endopeptidase homolog (*PHEX)* gene that are inherited in an X-linked dominant manner. These pathogenic variants lead to inappropriately normal or elevated circulating levels of the hormone fibroblast growth factor 23 (FGF23).^1^^,^^2^ Excess FGF23 impairs renal phosphate reabsorption and 1,25-dihydroxyvitamin D (1,25(OH)_2_D) production, resulting in chronic hypophosphatemia. In children, clinical manifestations of XLH include rickets, skeletal deformities, and impaired growth, whereas in adults, osteomalacic fractures, osteoarthritis, bone and joint pain, osteophytes, and enthesopathy are frequent manifestations.^2^

For approximately the past 40 yr, conventional therapy for XLH has consisted of active vitamin D combined with multiple daily doses of oral phosphate salts. However, such therapy has been associated with gastrointestinal upset, hypercalcemia, hypercalciuria, hyperparathyroidism, and nephrocalcinosis.^3^^-^^5^ Nephrocalcinosis is characterized by deposits of calcium phosphate or calcium oxalate within the interstitium of the kidney, usually in the medulla or less often the cortex.^6^ Among patients with XLH receiving conventional therapy, the reported prevalence of nephrocalcinosis varies widely,^4^^,^^5^^,^^7^^-^^18^ ranging from 20% to 80% in children^4^^,^^8^^,^^12^^,^^13^^,^^17^^,^^18^ and from 33% to 72% in adults.^7^^,^^8^^,^^10^^,^^11^^,^^17^^,^^18^ In most, but not all studies, nephrocalcinosis was observed only in those patients with XLH who had received conventional therapy,^4^^,^^5^^,^^8^^,^^12^^-^^15^^,^^17^ suggesting that nephrocalcinosis is not intrinsic to XLH but rather is a complication of oral phosphate and active vitamin D therapy. Indeed, nephrocalcinosis in patients with XLH receiving such therapy has been associated with vitamin D intoxication, higher doses of phosphate and/or active vitamin D, hypercalcemia, hypercalciuria, initiation of therapy at younger ages, and treatment length. However, other studies have shown no such associations.^4^^,^^5^^,^^11^^-^^13^^,^^15^^,^^17^ Although kidney function was reported to be normal in the majority of XLH patients with nephrocalcinosis, some had evidence of chronic kidney disease (CKD), including lower estimated glomerular filtration rates (eGFRs), interstitial nephritis, end-stage renal failure, and renal transplantation.^4^^,^^8^^,^^10^^,^^13^^,^^14^^,^^16^ XLH is a rare condition, thus most studies describing nephrocalcinosis and kidney function in this population are small (the largest published study to date had 52 patients^16^) and often single-center.^4^^,^^5^^,^^7^^-^^13^^,^^15^^,^^16^^,^^18^

Burosumab, a recombinant human monoclonal antibody that inhibits FGF23 activity, was first approved for the treatment of XLH in 2018 based on data from a series of clinical trials in affected children and adults.^19^^-^^22^ The XLH Disease Monitoring Program (XLH-DMP; NCT03651505) is a prospective, multinational, longitudinal outcomes study that examines the clinical, radiographic, and biochemical findings in children and adults with XLH and monitors the long-term safety of burosumab treatment, conventional therapy, or no therapy in this population. In the present study, we pooled baseline data from burosumab clinical trials (NCT02181764,^23^ NCT02526160,^19^ NCT02537431,^24^ NCT02163577,^20^ NCT02750618,^22^ NCT02915705^21^) and the XLH-DMP to describe nephrocalcinosis and renal function in a large number of patients with XLH from multiple sites and countries, allowing a more robust characterization of this population.

## Materials and methods

### Patients

The burosumab clinical trials and the XLH-DMP (as of data cutoff) were conducted in the USA (*n* = 283), Canada (*n* = 57), France (*n* = 33), Japan (*n* = 28), Chile (*n* = 26), Great Britain (*n* = 26), Brazil (*n* = 22), Korea (*n* = 14), Australia (*n* = 9), the Netherlands (*n* = 4), Ireland (*n* = 3), Italy (*n* = 3), Argentina (*n* = 1), and Sweden (*n* = 1). We included data from all patients with XLH who enrolled in a burosumab clinical trial and/or the XLH-DMP who underwent renal ultrasound examination at baseline, before exposure to burosumab.

The following clinical trials were conducted in adults with XLH: KRN23-001 (NCT02181764),^23^ CL303 (NCT02526160),^19^ and CL304 (NCT02537431).^24^ In studies CL303 and CL304, patients were ineligible for the trial if they had an eGFR of <45 mL/min, corrected serum calcium concentration of ≥10.8 mg/dL, or serum intact parathyroid hormone (iPTH) of ≥2.5-fold the upper limit of normal (ULN). In study KRN23-001 (NCT02181764), patients with uncontrolled hypertension or diabetes were also excluded (Table S1).

The following clinical trials were conducted in children with XLH: CL201 (NCT02163577)^20^ was carried out in children aged 5–12 yr, CL205 (NCT02750618)^22^ in children aged 1–4 yr, and CL301 (NCT02915705)^21^ in children aged 1–12 yr. Children with the following conditions were excluded in some of these studies: severe nephrocalcinosis (defined as a renal ultrasound score ≥3 in CL201 and renal ultrasound score of 4 in CL205 and CL301), hypocalcemia, or hypercalcemia. Study CL201 also excluded children with tertiary hyperparathyroidism and studies CL201 and CL301 excluded those whose height was ≥50th percentile for age and sex (Table S1). Enrollment in the XLH-DMP began on June 20, 2018; the data cutoff for the present report was April 30, 2020.

### Data

All data were collected at baseline. For patients who received burosumab, baseline was defined as the last non-missing assessment before first administration of burosumab. For patients who did not receive burosumab, baseline was defined as the last assessment available, either in the clinical trial in which they participated or at enrollment in the XLH-DMP, as appropriate. For those who participated in a clinical trial, only the baseline data from the clinical trial were used, rather than their measurements at XLH-DMP enrollment.

In children, height z-scores were determined using the United States Centers for Disease Control Macro: https://www.cdc.gov/nccdphp/dnpao/growthcharts/resources/sas.htm. For adults, height z-scores were calculated using the CDC height z-score macro for 20–yr-olds, since height z-score macros were not available on a population basis after this age, and since height z-scores are relatively stable beyond aged 20 yr (although there is some height loss later in life).^25^

Renal ultrasound examination was performed locally at the study sites at baseline. A central imaging facility, eResearch Technology (ERT, now Clario), provided training for the study sites and validated site equipment on which study ultrasounds were performed. Renal ultrasounds were graded centrally by a single reader at the central imaging facility on a 5-point scale,^26^ as follows:

1. Normal.
2. Faint hyperechogenic rim around the medullary pyramids.
3. More intense echogenic rim with echoes faintly filling the entire pyramid.
4. Uniformly intense echoes throughout the pyramid.
5. Stone formation: solitary focus of echoes at the tip of the pyramid.

Intra-reader reliability was assessed by randomly selecting 15 ultrasounds to be re-read. Nephrocalcinosis was defined as a renal ultrasound score ≥1. One patient had undergone renal transplantation of ~4 yr prior to enrollment in the XLH-DMP; the eGFR values for this patient at XLH-DMP baseline reflected the function of the transplanted kidney.

Conventional therapy consisted of oral administration of phosphate salts and active vitamin D. Duration of conventional therapy was based on patient recall, as recorded in the medical history, the treatment history, or prior medication study forms. Duration of conventional therapy was calculated from the reported start to the reported end of conventional therapy, or to baseline, whichever was most recent. Exposure to conventional therapy in some patients was not continuous; however, any gaps in therapy could not be fully determined and hence were not subtracted from the estimated total duration of therapy. Data regarding specific formulations of phosphate salts or active vitamin D were not available; baseline doses of phosphate salts were only available for 32 adults (10%) and 111 children (57%), and baseline doses of active vitamin D were only available for 45 adults (14%) and 93 children (47%).

Per protocol, blood and urine samples were collected after overnight fasting. In children aged <18 yr, eGFR was calculated using the creatinine-based CKiD U25 estimating equation from Pierce et al.^27^ In adults, eGFR was calculated using the creatinine-based Chronic Kidney Disease Epidemiology Collaboration equation.^28^ For both age groups, reduced eGFR was defined as <90 mL/min/1.73 m^2^. The tubular maximum reabsorption of phosphate per GFR (TmP/GFR) was calculated by Covance (now Labcorp Drug Development), which performed the biochemical testing, using the formula TmP/GFR = serum phosphate—(urinary phosphate/ urinary creatinine) × serum creatinine.^29^ Normal ranges for TmP/GFR were age-specific and based on Stark et al.^29^ All other age- and sex-specific normal ranges for children were based on CALIPER data.^30^ For adults, normal ranges were provided by Covance with the following exceptions. Normal ranges for serum alkaline phosphatase (ALP) and phosphorus concentrations were based on ranges provided by the Mayo Clinic (https://www.mayocliniclabs.com/). The upper limit for 1,25(OH)_2_D was set at 80 pg/mL, also in accordance with Mayo Clinic ranges. Normal ranges are shown in Table S2. Hypercalcemic hyperparathyroidism (also known as tertiary hyperparathyroidism) was defined as serum calcium above the ULN with normal or elevated iPTH levels.

### Statistics

The results presented here are descriptive and include mean, median, and SD by age group (children and adults) and nephrocalcinosis (presence or absence). The number and percentage of patients whose biochemical values exceeded the ULN and those that were below the lower limit of normal (LLN) for the various biochemical values were assessed. Two-sided *t*-tests were used to compare mean values in children with nephrocalcinosis vs children without nephrocalcinosis and adults with nephrocalcinosis vs adults without nephrocalcinosis. For serum phosphorus and TmP/GFR, chi-squared tests were used to compare the percentage of patients below the LLN in children with nephrocalcinosis vs children without nephrocalcinosis and adults with nephrocalcinosis vs adults without nephrocalcinosis. Normal ranges are shown in Table S2. Serum phosphorus z-scores for children were calculated using CALIPER data^30^ with a constant SD of 0.61 mg/dL across ages.

As an exploratory analysis, a series of univariate logistic regressions were performed for the entire population and separately for children and adults; the presence or absence of nephrocalcinosis was the dependent variable. The following independent variables were tested individually: demographics (age, sex), treatment characteristics (durations of conventional therapy, and in the subset of children for whom data were available, doses of conventional therapy), serum biochemistry (serum total calcium, phosphorus, 25OHD, 1,25(OH)_2_D, ALP, and iPTH), serum phosphorus z-score, height z-score, kidney function (eGFR, and TmP/GFR), and 24-h urinary calcium excretion.

## Results

### Patient disposition

This study included a total of 514 patients, 196 children and 318 adults, for whom baseline renal ultrasound scores and select biochemical data were available before administration of burosumab. Of these, 284 patients were first enrolled in a burosumab clinical trial, and 230 were first enrolled in the XLH-DMP (Table S1). During enrollment in the burosumab clinical trials, the following patients met exclusion criteria for kidney function and were, therefore, not included in the present analysis: 3 adults with eGFR <60 mL/min, 5 children with serum creatinine greater than the ULN, and 3 children with renal ultrasound nephrocalcinosis score ≥3.

### Patient demographics and baseline characteristics

In children, mean (SD) age at reported XLH diagnosis was 1.6 (1.9) yr, and mean age at baseline was 7.6 (4.0) yr (Table 1). The majority were female (112/196, 57%) and resided in the USA (113/196, 58%) or Canada (28/196, 14%). Mean (SD) height z-score at baseline was −1.9 (1.2). In adults, mean (SD) age at reported XLH diagnosis was 9.4 (15.3) yr, mean age at baseline was 40.3 (13.1) yr, and mean height z-score at baseline was −2.3 (1.7) (Table 1). The majority of adults were female (232/318, 73%) and resided in the USA (172/318, 54%) or Canada (29/318, 9%).

**Table 1:** Baseline demographics and disease characteristics.

	Children			Adults		
​	**No NC** ***n* = 152​**	**NC** ***n* = 44​**	**Total** ***n* = 196​**	**No NC** **N = 196​**	**NC** ***n* = 122​**	**Total** ***n* = 318**
**Age at XLH Diagnosis, yr** **Mean (SD)** **Median**	*n* = 145 1.7 (2.0) 1.2	*n* = 43 1.4 (1.8) 0.6	*n* = 188 1.6 (1.9) 1.2	*n* = 174 11.0 (16.8) 2.8	*n* = 96 6.5 (11.6) 2.2	*n* = 270 9.4 (15.3) 2.2
**Age at baseline, yr** **Mean (SD)** **Median**	7.0 (4.1) 6.7	9.3 (3.0) 9.6	7.6 (4.0) 7.8	41.4 (13.6) 39.1	38.5 (12.0) 38.8	40.3 (13.1) 39.0
**Female, *n* (%)**	91 (60)	21 (48)	112 (57)	152 (78)	80 (66)	232 (73)
**Race, *n* (%)**						
**White**	115 (76)	35 (80)	150 (77)	146 (75)	90 (74)	236 (74)
**Black**	8 (5)	1 (2)	9 (5)	4 (2)	1 (1)	5 (2)
**Asian**	10 (7)	0 (0)	10 (5)	16 (8)	23 (19)	39 (12)
**Other/Unknown**	19 (13)	8 (18)	27 (14)	30 (15)	8 (7)	38 (12)
**Region, *n* (%)**						
**USA**	88 (58)	25 (57)	113 (58)	104 (53)	68 (56)	172 (54)
**Canada**	22 (15)	6 (14)	28 (14)	23 (12)	6 (5)	29 (9)
**Europe**	12 (8)	10 (23)	22 (11)	26 (13)	22 (18)	48 (15)
**Asia-Pacific**	15 (10)	1 (2)	16 (8)	14 (7)	21 (17)	35 (11)
**Latin America**	15 (10)	2 (5)	17 (9)	29 (15)	5 (4)	34 (11)
**Height z-score** **Mean (SD)** **Median**	*n* = 144 −2.0 (1.1) −2.0	*n* = 44 −1.8 (1.3) −1.6	*n* = 188 −1.9 (1.2) −1.9	*n* = 189 −2.3 (1.7) −2.3	*n* = 122 −2.4 (1.6) −2.2	*n* = 311 −2.3 (1.7) −2.3

Abbreviation: NC, nephrocalcinosis. Nephrocalcinosis was defined as a renal ultrasound score ≥ 1. The number of patients in each group was listed if there were any missing data.

**Figure 1:**
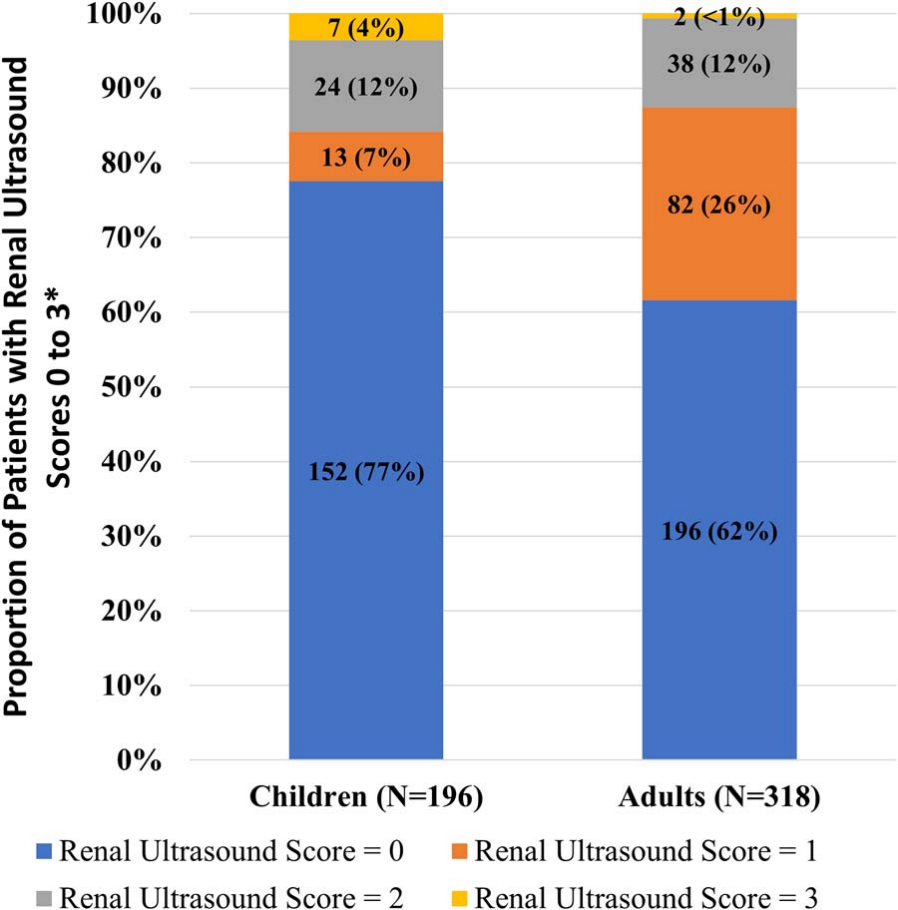
Prevalence of nephrocalcinosis in children and adults with XLH. ^*^No patients had a renal ultrasound score of 4 or 5.

At baseline, nephrocalcinosis was detected in 44 of 196 children (22%) (Figure 1). Children with nephrocalcinosis were slightly older at baseline than those without: mean (SD) 9.3 (3.0) yr vs 7.0 (4.1) yr, respectively (Table 1). There were no marked differences with regards to age at diagnosis, gender, race, region, and mean height z-scores at baseline between the 2 groups. Mean (SD) height z-score was −1.8 (1.3) in those with nephrocalcinosis and −2.0 (1.1) in those without.

Nephrocalcinosis was detected in 122 of 318 adults (38%) (Figure 1). Adults with nephrocalcinosis were younger at XLH diagnosis than those without nephrocalcinosis: 6.5 (11.6) yr and 11.0 (16.8) yr. No other marked differences were apparent between the 2 groups.

### Duration of conventional therapy

At baseline, 190 of 196 children (97%) were receiving or had received prior therapy with both phosphate and active vitamin D (Table 2), with the remainder being untreated. Of the 318 adults, 281 (88%) had received both phosphate and active vitamin D (Table 2), 9 (3%) had received only phosphate, 10 (3%) only active vitamin D, and 18 (6%) no prior conventional therapy.

**Table 2:** Conventional therapy in children and adults.

	Children			Adults		
​	**No NC** ***n* = 152**	**NC** ***n* = 44**	**Total** ***n* = 196**	**No NC** **N = 196**	**NC** ***n* = 122**	**Total** ***n* = 318**
**Prior conventional therapy, *n* (%)**	146 (96)	44 (100)	190 (97)	179 (91)	121 (99)	300 (94)
**Phosphate only, *n* (%)**	0	0	0	6 (3)	3 (2)	9 (3)
**Active vitamin D only, *n* (%)**	0	0	0	8 (4)	2 (2)	10 (3)
**Phosphate and active vitamin D, *n* (%)**	146 (96)	44 (100)	190 (97)	165 (84)	116 (95)	281 (88)
**No prior conventional therapy, *n* (%)**	6 (4)	0	6 (3)	17 (9)	1 (1)	18 (6)
**Age phosphate initiated, yr** **Mean (SD)** **Median**	*n* = 142 2.2 (2.1) 1.9	*n* = 43 1.8 (2.0) 1.1	*n* = 185 2.1 (2.1) 1.9	*n* = 119 12.8 (15.4) 5.0	*n* = 72 20.1 (17.8) 18.4	*n* = 191 15.6 (16.7) 7.0
**Age active vitamin D initiated, yr** **Mean (SD)** **Median**	*n* = 140 2.3 (2.4) 1.9	*n* = 41 1.8 (2.0) 1.0	*n* = 181 2.2 (2.3) 1.9	*n* = 117 13.7 (15.9) 5.00	*n* = 80 20.4 (17.0) 21.1	*n* = 197 16.5 (16.7) 8.0
**Duration of phosphate therapy, yr** **Mean (SD)** **Median**	*n* = 148 4.3 (3.6) 3.4	*n* = 43 7.4 (3.8) 7.8	*n* = 191 5.0 (3.8) 4.0	*n* = 160 13.2 (13.5) 10.0	*n* = 76 10.9 (10.6) 10.0	*n* = 236 12.5 (12.6) 10.0
**Duration of active vitamin D therapy, yr** **Mean (SD)** **Median**	*n* = 146 4.3 (3.6) 3.4	*n* = 41 7.4 (3.8) 7.8	*n* = 187 5.0 (3.8) 4.0	*n* = 166 12.3 (12.8) 9.0	*n* = 84 11.4 (11.2) 9.1	*n* = 250 12.0 (12.2) 9.0

Abbreviation: NC = nephrocalcinosis. Nephrocalcinosis was defined as a renal ultrasound score ≥ 1.

The number of patients in each group was listed if there were any missing data.

All patients with nephrocalcinosis had previously received conventional therapy, with the sole exception of a 45-yr-old woman who was diagnosed with XLH at 33 yr of age and had enrolled in the XLH-DMP without prior treatment of any kind. Her height at DMP baseline was 125 cm (z-score −5.8), indicating extreme short stature. Her baseline renal ultrasound score was 1, indicating mild nephrocalcinosis. PTH level was elevated (100 pg/mL); serum and urine calcium levels were not available. Apart from the diagnosis of XLH, no additional, relevant medical history was reported.

Children initiated phosphate therapy at a mean (SD) age of 2.1 (2.1) yr and active vitamin D at 2.2 (2.3) yr (Table 2). Mean (SD) duration of phosphate therapy and active vitamin D therapy was 7.4 (3.8) yr for children with nephrocalcinosis and 4.3 (3.6) yr for those without (Table 2).

Adults reported initiating phosphate therapy at a mean (SD) age of 15.6 (16.7) yr and active vitamin D at 16.5 (16.7) yr (Table 1). Mean duration of phosphate and active vitamin D was similar in adults with nephrocalcinosis compared to those without (a difference of 2.3 and 0.9 yr, respectively).

### Biochemistry

**Figure 2:**
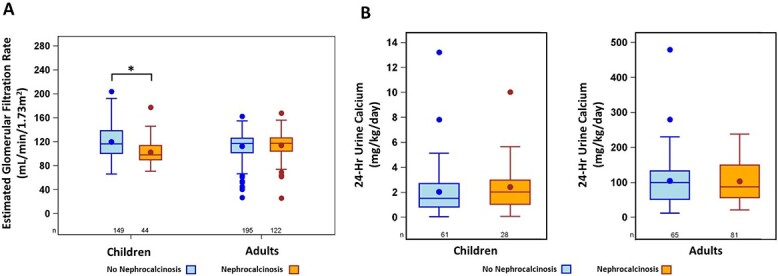
Estimated GFR and urine calcium excretion in children and adults. (A) Estimated GFR in children and adults. Circles within the shaded bars indicate means. ^*^p<.001. Includes 2 infants with eGFR <90 mL/min/1.73 m^2^, which was within normal levels for this age range. Two outliers with high estimates of GFR may skew the data. (B). Twenty-four-hour urine calcium excretion in children and adults. For the 3 children with abnormally high 24-h urine calcium excretion, the 2-h urine calcium excretion to creatinine ratio was in the normal range. However, data on 24-h creatinine excretion were not available, and outlier values could not be further validated.

The differences in biochemical results among participants with and without nephrocalcinosis are shown in Table 3 and Figure 2. In children, hypophosphatemia at baseline measurement was present in nearly all children with nephrocalcinosis (42/44, 96%) and in those without (148/150, 99%). Children with nephrocalcinosis had lower mean TmP/GFR (*p*<.05), serum 1,25(OH)_2_D (*p*<.05), and eGFR (*p*<.001), as well as higher serum calcium concentrations (*p*<.05) (Table 3**,**  Figure 2). The lower mean eGFR in those with nephrocalcinosis might have been driven by several high outlier values in those without nephrocalcinosis. Accordingly, a sensitivity analysis was performed in which eGFR values >190 mL/min/1.73 m^2^ from each group were excluded (only 2 children without nephrocalcinosis exceeded this level and were excluded; Figure S1 ). Mean (SD) eGFR remained significantly lower in children with nephrocalcinosis than that in those without (*p*<.001). Furthermore, eGFR was <90 mL/min/m^2^ in 11 of 44 (25%) children with nephrocalcinosis compared with 16 of 149 (11%) in those without nephrocalcinosis. There were no differences in mean serum phosphorus levels, serum phosphorus z-scores, ALP, iPTH, 25OHD, 1,25(OH)_2_D, or 24-h urine calcium in children with nephrocalcinosis vs those without.

**Table 3:** Baseline biochemical studies.

	Children			Adults		
	**No NC** *n* = 152	**NC** *n* = 44	**Total** *n* = 196	**No NC** *n* = 196	**NC** *n* = 122	**Total** *n* = 318
**Serum phosphorus** **Patients below LLN, *n* (%)**	*n* = 150 148 (99)	*n* = 44 42 (96)	*n* = 194 190 (98)	*n* = 195 155 (79)	*n* = 122 109 (89)	*n* = 317 264 (83)
	Below LLN: *p*=.19			**Below LLN: *p*<.05**		
**mg/dL, mean (SD)** **median**	2.5 (0.5) 2.5	2.4 (0.5) 2.3	2.5 (0.5) 2.5	2.2 (0.4) 2.2	2.0 (0.4) 2.0	2.1 (0.4) 2.1
	Mean: *p*=.06			**Mean: *p*<.01**		
**Z-score, mean (SD)** **median**	−4.2 (0.8) −4.3	−4.2 (1.0) −4.5	−4.2 (0.8) −4.4			
**TmP/GFR** **Patients below LLN, *n* (%)**	*n* = 75 73 (97)	*n* = 31 31 (100)	*n* = 106 104 (98)	*n* = 68 65 (96)	*n* = 90 87 (97)	*n* = 158 152 (96)
	Below LLN: *p*=.52			Below LLN: *p*=.73		
**mg/dL, mean (SD)** **median**	2.1 (0.4) 2.2	1.9 (0.4) 1.9	2.1 (0.4) 2.0	1.7 (0.4) 1.7	1.7 (0.4) 1.7	1.7 (0.4) 1.7
	**Mean: *p*<.05**			Mean: *p*=.24		
**Serum calcium** **Patients above ULN, *n* (%)** **mg/dL, mean (SD)** **median**	*n* = 110 5 (5) 9.6 (0.4) 9.6	*n* = 35 6 (17) 9.9 (0.5) 9.8	*n* = 145 11 (8) 9.7 (0.5) 9.7	*n* = 99 3 (3) 9.3 (0.5) 9.2	*n* = 100 0 (0) 9.2 (0.5) 9.1	*n* = 199 3 (2) 9.2 (0.5) 9.1
	**Mean: *p*<.05**			Mean: *p*=.13		
**Serum iPTH** **Patients above ULN, *n* (%)** **pg/mL, mean (SD)** **median**	*n* = 147 31 (21) 50.1 (34.0) 42.1	*n* = 44 10 (23) 49.0 (30.4) 44.1	*n* = 191 41 (22) 49.9 (33.1) 42.5	*n* = 195 102 (52) 85.3 (50.1) 76.0	*n* = 121 76 (63) 94.3 (73.6) 82.0	*n* = 316 178 (56) 88.8 (60.2) 78.0
	Mean: *p*=.84			Mean: *p*=.20		
**ALP** **Patients above ULN, *n* (%) U/L,** **mean (SD)** **median**	*n* = 147 121 (82) 474 (167) 467	*n* = 44 34 (77) 431 (122) 438	*n* = 191 155 (81) 464 (159) 464	*n* = 104 52 (50) 120 (47) 112	*n* = 100 46 (46) 132 (87) 107	*n* = 204 98 (48) 126 (70) 110
	Mean: *p*=.11			Mean: *p*=.20		
**1,25(OH)**_**2**_ **D** **Patients below LLN** **pg/mL, mean (SD)** **median**	*n* = 141 75 (53) 46.4 (18.9) 43.5	*n* = 42 29 (69) 36.9 (16.9) 35.0	*n* = 183 104 (57) 44.2 (18.7) 41.4	*n* = 182 32 (18) 40.6 (16.6) 39.1	*n* = 115 36 (31) 36.7 (17.1) 34.5	*n* = 297 68 (23) 39.1 (16.8) 37.6
	**Mean: *p*<.05**			**Mean: *p*<.05**		
**25OHD** **Patients <30 ng/mL, *n* (%)** **Patients <20 ng/mL, *n* (%)** **ng/mL, mean (SD)** **median**	*n* = 94 43 (46) 9 (10) 31.2 (9.7) 30.8	*n* = 31 10 (32) 2 (7) 32.6 (10.2) 32.4	*n* = 125 53 (42) 11 (9) 31.6 (9.8) 31.3	*n* = 68 49 (72) 22 (32) 24.2 (8.7) 24.1	*n* = 90 59 (66) 30 (33) 25.8 (12.1) 24.1	*n* = 158 108 (68) 52 (33) 25.1 (10.8) 24.1
	Mean: *p*=.51			Mean: *p*=.36		

Abbreviation:ALP, alkaline phosphatase; iPTH, intact parathyroid hormone; LLN, lower limit of normal; NC: nephrocalcinosis; TmP/GFR, tubular maximum for phosphate reabsorption per GFR; ULN, upper limit of normal.

Nephrocalcinosis was defined as a renal ultrasound score ≥1. Two-sided *t*-tests were used to compare mean values, and chi-squared tests were used to compare the percentage of patients below the LLN in children with nephrocalcinosis vs children without nephrocalcinosis and adults with nephrocalcinosis vs adults without nephrocalcinosis. Normal ranges are shown in Supplemental Table 2

In adults, hypophosphatemia at baseline measurement was significantly more prevalent in those with nephrocalcinosis (109/122, 89%) vs those without (155/195, 79%, *p*<.05). Mean serum phosphorus (*p*<.01) and serum 1,25(OH)_2_D levels (*p*<.05) were significantly lower among patients with nephrocalcinosis than those without. There were no significant differences in TmP/GFR, mean serum calcium, 24-h urine calcium, serum iPTH, ALP, 25OHD, or eGFR between the 2 groups.

### Glomerular filtration rate

Tables S3 and S4 provide demographic and biochemical data for children and adults based on eGFR. Estimated GFR was <90 mL/min/1.73 m^2^ in 27 of 193 children (14%). Among these patients, eGFR was 80–90 mL/min/1.73 m^2^ in 19 of 193 children (9.8%) and 70–80 mL/min/1.73 m^2^ in 6 of 193 children (3%). No child had an eGFR below 70 mL/min/1.73 m^2^ except for 2 infants aged 1 and 3 mo, when GFR normally is lower and therefore within the normal range for age (40–60 mL/min/1.73 m^2^; Table 4). Of the 27 children with eGFR <90 mL/min/1.73 m^2^, nephrocalcinosis was observed in 11 children (41%), a higher prevalence than that observed in children with eGFR ≥90 mL/min/1.73 m^2^ (33/166, 20%). No differences in other demographic or biochemical characteristics were apparent in those with eGFR <90 mL/min/1.73 m^2^ compared to those with eGFR ≥90 mL/min/1.73 m^2^.

**Table 4:** Estimated GFR ranges in patients with and without nephrocalcinosis.

**eGFR**	**Chronic kidney disease stages**	**Children**			**Adults**		
		**No NC** ***n* = 149**	**NC** ***n* = 44**	**Total** ***n* = 193**	**No NC** ***n* = 195**	**NC** ***n* = 122**	**Total** ***n* = 317**
**≥90**	Normal, *n* (%)	133 (88)	33 (75)	166 (85)	168 (86)	107 (88)	275 (87)
**60–89**	Stage 2, *n* (%)	16 (11)^a^	11 (25)	27 (14)^a^	21 (11)	14 (12)	35 (11)
**30–59**	Stage 3, *n* (%)	0 (0)	0 (0)	0 (0)	5 (3)	0 (0)	5 (2)
**15–29**	Stage 4, *n* (%)	0 (0)	0 (0)	0 (0)	1 (1)	1 (1)	2 (1)

Abbreviation: eGFR, estimated glomerular filtration rate; NC, nephrocalcinosis. Nephrocalcinosis was defined as a renal ultrasound score ≥ 1.

a Includes 2 infants with eGFR <90 mL/min/1.73 m^2^, which is within the normal range of values for this age.

Estimated GFR was <90 mL/min/1.73 m^2^ in 42 of 317 (13%) adults, 35 (11%) with eGFR 60–89 mL/min/1.73 m^2^ (stage 2 CKD), 5 (2%) with eGFR 30–59 mL/min/1.73 m^2^ (stage 3 CKD), and 2 (1%) with eGFR 15–29 mL/min/1.73 m^2^ (stage 4 CKD). The percentage with eGFR <90 mL/min/1.73 m^2^ was similar among adults with nephrocalcinosis (15/122, 12%) and those without (27/195; 14%; Table 4). Of those with reduced eGFR <90 mL/min/1.73 m^2^, nephrocalcinosis was present in 15 of 42 (36%), a percentage similar to those with a normal eGFR, 107 of 275 (39%).

A history of parathyroidectomy was reported in 10 of 317 adults (3%). Five of these adults had stage 3–4 CKD, whereas 5 had eGFRs in the normal range. The proportion of patients who had parathyroidectomy was similar among those with nephrocalcinosis (4/122, 3%) and without (6/195, 3%). None of the children enrolled in this study were reported to have parathyroidectomy.

Based on baseline laboratory values, hypercalcemic hyperparathyroidism was seen in 11 of 145 children (7.6%) and 3 of 199 adults (1.5%). The prevalence of nephrocalcinosis in children with hypercalcemic hyperparathyroidism, 6/11 (55%), was more than double that in children without hypercalcemic hyperparathyroidism, 38/187 (20%). None of the 3 adults with hypercalcemic hyperparathyroidism had nephrocalcinosis.

One adult with XLH developed end-stage renal disease in her early 30s and underwent renal transplantation, which was complicated by chronic rejection requiring a second transplant in her 50s, before she enrolled in the XLH-DMP. Per patient report, the patient was born with a solitary kidney, and end-stage renal disease was attributed to treatment with supraphysiological doses of phosphate and vitamin D, which resulted in hypercalcemia, hyperphosphatemia, tertiary hyperparathyroidism, and nephrocalcinosis. Baseline ultrasound score for the transplanted kidney was 0, indicating no nephrocalcinosis.

### Regression analysis

As an exploratory analysis, multiple separate univariate logistic regressions were carried out to identify factors that associate with nephrocalcinosis. We observed no significant associations between the presence or absence of nephrocalcinosis and duration of therapy, baseline eGFR, serum phosphorus, serum calcium, serum iPTH, 24-h urine calcium excretion, TmP/GFR, ALP, phosphorus z-scores, or height z-scores (neither for the group as a whole nor in either children or adults when analyzed separately). In those children for whom data on the daily dose of oral phosphate or active vitamin D were available (*n* = 88), no significant associations were detected between presence/absence of nephrocalcinosis and doses of either phosphate or active vitamin D.

## Discussion

We describe nephrocalcinosis and kidney function in 514 well-characterized children and adults with XLH, the vast majority of whom had received conventional therapy from childhood for a duration as long as 57 yr. These data represent the largest XLH cohort described to date, with all data collected before any patient received burosumab. Nephrocalcinosis was detected in 22% of children and 38% of adults. Of interest, eGFR was mildly reduced (<90 mL/min/1.73 m^2^) in 14% of children and in the same proportion of adults. Two adults (1%) had stage 4 CKD, whereas none of the children had CKD greater than stage 2. A single adult patient developed end-stage renal failure in her early 30s that was associated with hypercalcemia and nephrocalcinosis and underwent renal transplantation, which was complicated by chronic rejection. She underwent a second renal transplant in her 50s, before enrollment in the XLH-DMP.

In the next largest, single-center retrospective report of 52 adults with XLH who were observed between 1934 and 1989,^16^ 3 patients developed end-stage renal failure secondary to vitamin D toxicity, but additional details regarding the kidney, including the presence of nephrocalcinosis, were not described. The prevalence of nephrocalcinosis we observed (22% of children and 38% of adults) was within the ranges reported in much smaller studies of 5 to 25 patients.^4^^,^^5^^,^^7^^,^^8^^,^^10^^-^^13^^,^^15^^,^^17^^,^^18^

To explore risk factors for the development of nephrocalcinosis, we examined the relationship between nephrocalcinosis and patient demographics, treatment characteristics, and disease characteristics, but we identified no significant associations. Although the number of children with hypercalcemic hyperparathyroidism was small (7.6%), nephrocalcinosis was more than twice as prevalent in those with hypercalcemic hyperparathyroidism than in those without, highlighting the need to monitor for and mitigate hyperparathyroidism-induced hypercalcemia to the degree possible, as outlined in previously described strategies.^31^

In nearly all published studies, nephrocalcinosis was absent in treatment-naïve patients with XLH.^4^^,^^5^^,^^12^^,^^14^^,^^15^^,^^17^ Yet, in one study, 4 of 23 adults had nephrocalcinosis despite lack of prior treatment with oral phosphate during childhood (treatment with phosphate during adulthood was not detailed).^8^ Notably, all of these patients were treated with calcitriol, although the duration of use was not reported.^8^ In the present study, 24 of 514 patients (5%) reported absence of prior conventional therapy, only one of whom had evidence of mild nephrocalcinosis. In this 45-yr-old, treatment-naïve patient with XLH, serum PTH was high and 1,25(OH)_2_D was within the normal range. Whether nephrocalcinosis in this patient was related to XLH or another cause remains unclear. Thus, although it seems likely, it cannot be said definitively that nephrocalcinosis is solely a complication of conventional therapy of XLH.

The strength of our study is the large number of patients (exceeding the number of patients in the published literature by nearly 10-fold) and multinational participation in this study. However, our study has several limitations. Because severe nephrocalcinosis was an exclusion criterion in 2 of the burosumab clinical trials, selection bias could have resulted in an underestimation of the prevalence of nephrocalcinosis and reduced eGFR in the broader population of XLH patients. Similarly, the clinical trials also had varying exclusion criteria regarding high serum calcium and PTH levels, likely influencing the prevalence of hypercalcemic hyperparathyroidism that we observed. For adults, the age of diagnosis and duration of conventional therapy may have been subject to recall bias. Data on the doses of oral phosphate and/or active vitamin D were not available for most participants, and changes in dose over time, as well as gaps in treatment may have influenced nephrocalcinosis development. Additionally, precise information on the formulation of phosphate salts and active vitamin D was not available. The severity of nephrocalcinosis was subject to reader interpretation; however, observer bias was minimized by use of a single reader in the present study. Data for some of the biochemical tests were not available for all patients, thus the incidence of some abnormal values might be underestimated. For example, although iPTH was measured in nearly every patient at baseline, serum calcium was measured in only 145/196 children (74%) and 199/318 adults (63%), thus limiting the ability to detect hypercalcemia or hypercalcemic hyperparathyroidism. Twenty-four hour urine calcium was measured in only 89/196 children (45%) and 146/318 (46%) adults at baseline. Finally, the time interval between the last doses of conventional therapy and biochemical testing was not known. In the clinical trials, the period of washout from conventional therapy ranged from 14 d to 2 yr for adults, and from 7 to14 d for children; those in the XLH-DMP had no washout period. This could render it more difficult to detect association(s) between nephrocalcinosis and certain biochemical test values, such as serum phosphorus, serum calcitriol, serum calcium and urine calcium excretion, which can be affected by conventional therapy.

The findings brought forward in the present study raise a number of important questions that provide a foundation for generating hypotheses related to kidney health in XLH. Compared to those without nephrocalcinosis, both children and adults with nephrocalcinosis had lower mean serum 1,25(OH)_2_D levels, and adults had lower serum phosphorus levels. These findings raise the question whether serum FGF23 levels were higher and disease severity greater in patients with nephrocalcinosis. In the present study, serum FGF23 levels were not available to interrogate this hypothesis; furthermore, whether 1,25(OH)_2_D levels reflect disease severity in untreated patients has not been specifically examined. Further study is also required to better understand the relationships among nephrocalcinosis, disease characteristics, and treatment strategies (with detailed capture of treatment regimens including doses, durations and specific preparations of the various potential therapies). The present report also does not directly address the question of the potential long-term renal consequences of nephrocalcinosis. We did, however, observe that eGFR was <90 mL/min/1.73 m^2^ in one-quarter of the children with nephrocalcinosis, a prevalence greater than in those without (11%). No such difference was seen in adults; the reason for this age-related difference also requires further study.

Patients enrolled in the XLH-DMP will be monitored prospectively for up to 10 yr, including with renal ultrasound examinations, to attempt to address the questions raised in the present baseline report. Thus, the XLH-DMP will provide additional prospective information on the natural history of XLH with respect to renal complications over an extended period of time.

## Supplementary Material

Supplemental_Information_zjae127

## Data Availability

Requests for individual de-identified participant data and the clinical study report from this study will be available after product approval to researchers providing a methodologically sound proposal that is in accordance with the Ultragenyx data sharing commitment. To gain access, data requestors will need to sign a data access and use agreement. Data will be shared via secured portal.
