# Supplementary material for: Nephrocalcinosis and kidney function in children and adults with X-linked hypophosphatemia: baseline results from a large longitudinal study
Source: J Bone Miner Res. 2024 Aug 16;39(10):1493–502. doi: 10.1093/jbmr/zjae127 (PMC11425691; doi:10.1093/jbmr/zjae127)

**Supplemental Tables**

**Supplemental Table 1. Patient disposition**

| **Parent Study** | **Enrolled in Parent Study (N)** | **Unique patients with pre-burosumab baselines and renal ultrasound scores (N, %)** | **Rationale** |
| --- | --- | --- | --- |
| KRN23-001  NCT02181764 | 18 | 18, 4% |  |
| UX023-CL303  NCT02526160 | 134 | 127, 25% | - 4 patients enrolled and took burosumab in KRN23-001. Pre-burosumab baseline and renal ultrasound scores captured in KRN23-001 were used here. - 3 patients enrolled and took burosumab in a previous clinical trial that did not collect renal ultrasound scores, so no pre-burosumab scores were available. |
| UX023-CL304  NCT02537431 | 14 | 13, 2% | - 1 patient enrolled and took burosumab in KRN23-001. Pre-burosumab baseline and renal ultrasound score captured in KRN23-001 was used here. |
| UX023-CL201  NCT02163577 | 52 | 52, 10% |  |
| UX023-CL205  NCT02750618 | 13 | 13, 2% |  |
| UX023-CL301  NCT02915705 | 61 | 61, 12% |  |
| XLH-DMP  NCT03651505 | 594 | 230, 45% | - 125 patients enrolled and took burosumab in a previous clinical trial. Pre-burosumab data and renal ultrasound scores captured in the previous trials were used here. - 22 patients enrolled and took burosumab in a previous clinical trial that did not collect renal ultrasound scores, so no pre-burosumab scores were available. - 144 patients took commercial burosumab before enrolling in the XLH-DMP, so no pre-burosumab data were available. - 73 patients did not have renal ultrasound scores available from XLH-DMP baseline. |

**Supplemental Table 2. Normal lab ranges**

| **Lab** | **Sex** | **Age** | **Normal Range** | **Unit** | **Source** |
| --- | --- | --- | --- | --- | --- |
| **1,25(OH)_2_D** | Both | 0-1 y | 32.1-196 | pg/mL | CALIPER |
|  |  | 1-3 y | 47.1-151 |  | CALIPER |
|  |  | 3-18 y | 45-102 |  | CALIPER |
|  |  | ≥18 y | 27.5-80 |  | Covance |
| **25-hydroxyvitamin D** | Both | 5-14 d | 1.7-34 | ng/mL | CALIPER |
|  |  | 15 d-3 mon | 6.2-40.5 |  | CALIPER |
|  |  | 3 mon-1 y | 7.0-47.3 |  | CALIPER |
|  |  | 1-9 y | 13.2-54.9 |  | CALIPER |
|  |  | 9-14 y | 12.7-46.5 |  | CALIPER |
|  |  | 14-18 y | 4.8-42.3 |  | CALIPER |
|  |  | ≥18 y | ≥29.0 |  | Covance |
| **ALP** | Female | 0-15 d | 90-273 | U/L | CALIPER |
|  |  | 15 d-1 y | 134-518 |  | CALIPER |
|  |  | 1-10 y | 156-369 |  | CALIPER |
|  |  | 10-13 y | 141-460 |  | CALIPER |
|  |  | 13-15 y | 62-280 |  | CALIPER |
|  |  | 15-17 | 54-128 |  | CALIPER |
|  |  | 17-18 | 48-95 |  | CALIPER |
|  |  | ≥18 y | 25-104 |  | Mayo |
|  | Male | 0-15 d | 90-273 |  | CALIPER |
|  |  | 15 d-1 y | 134-518 |  | CALIPER |
|  |  | 1-10 y | 156-369 |  | CALIPER |
|  |  | 10-13 y | 141-460 |  | CALIPER |
|  |  | 13-15 y | 127-517 |  | CALIPER |
|  |  | 15-17 | 89-365 |  | CALIPER |
|  |  | 17-18 | 59-164 |  | CALIPER |
|  |  | ≥18 y | 40-129 |  | Mayo |
| **Creatinine** | Female | 0-15 d | 0.4-1.1 | mg/dL | CALIPER |
|  |  | 15 d-1 y | 0.3-0.5 |  | CALIPER |
|  |  | 1-4 y | 0.4-0.6 |  | CALIPER |
|  |  | 4-7 y | 0.4-0.7 |  | CALIPER |
|  |  | 7-12 y | 0.5-0.7 |  | CALIPER |
|  |  | 12-15 y | 0.6-0.8 |  | CALIPER |
|  |  | 15-18 y | 0.7-1.0 |  | CALIPER |
|  |  | 18-50 y | 0.4-1.1 |  | Covance |
|  |  | 50-70 y | 0.4-1.1 |  | Covance |
|  |  | 70-80 y | 0.4-1.2 |  | Covance |
|  |  | ≥80 y | 0.4-1.4 |  | Covance |
|  | Male | 0-15 d | 0.4-1.1 |  | CALIPER |
|  |  | 15 d-1 y | 0.3-0.5 |  | CALIPER |
|  |  | 1-4 y | 0.4-0.6 |  | CALIPER |
|  |  | 4-7 y | 0.4-0.7 |  | CALIPER |
|  |  | 7-12 y | 0.5-0.7 |  | CALIPER |
|  |  | 12-15 y | 0.6-0.8 |  | CALIPER |
|  |  | 15-18 y | 0.6-0.9 |  | CALIPER |
|  |  | 18-50 y | 0.5-1.2 |  | Covance |
|  |  | 50-70 y | 0.5-1.3 |  | Covance |
|  |  | 70-80 y | 0.5-1.5 |  | Covance |
|  |  | ≥80 y | 0.5-1.6 |  | Covance |
| **Serum calcium** | Both | 0-1 y | 8.5-11.0 | mg/dL | CALIPER |
|  |  | 1-18 y | 9.2-11 |  | CALIPER |
|  |  | ≥18 y | 8.3-10.6 |  | Covance |
| **iPTH** | Both | 6 d-1 y | 0.68-9.39 | pmol/L | CALIPER |
|  |  | 1-9 y | 1.72-6.68 |  | CALIPER |
|  |  | 9-17 y | 2.32-9.28 |  | CALIPER |
|  |  | 17-18 y | 1.7-6.4 |  | CALIPER |
|  |  | ≥18 y | 1.48-7.63 |  | Covance |
| **Serum phosphorus** | Female | 0-15 d | 5.6-10.5 | mg/dL | CALIPER |
|  |  | 15 d-1 y | 4.8-8.4 |  | CALIPER |
|  |  | 1-5 y | 4.3-6.8 |  | CALIPER |
|  |  | 5-13 y | 4.1-5.9 |  | CALIPER |
|  |  | 13-16 y | 3.2-5.5 |  | CALIPER |
|  |  | 16-18 y | 2.9-5 |  | CALIPER |
|  |  | ≥18 y | 2.5-4.5 |  | Mayo |
|  | Male | 0-15 d | 5.6-10.5 |  | CALIPER |
|  |  | 15 d-1 y | 4.8-8.4 |  | CALIPER |
|  |  | 1-5 y | 4.3-6.8 |  | CALIPER |
|  |  | 5-13 y | 4.1-5.9 |  | CALIPER |
|  |  | 13-16 y | 3.5-6.2 |  | CALIPER |
|  |  | 16-18 y | 2.9-5.0 |  | CALIPER |
|  |  | ≥18 y | 2.5-4.5 |  | Mayo |
| **TmP/GFR** | Both | 2-12 y | 3.4-5.8 | mg/dL | Stark et al |
|  |  | ≥18 y | 2.5-4.2 |  | Stark et al |

ALP: alkaline phosphatase. iPTH: intact parathyroid hormone. TmP/GFR: tubular maximum for phosphate reabsorption per GFR.

Normal ranges for TmP/GFR were age-specific and based on Stark et al^29^. Age- and sex-specific normal ranges for children were based on CALIPER data^30^. Normal ranges for adult lab values were provided by Covance, with the following exceptions. Normal ranges for alkaline phosphatase (ALP) and serum phosphorus concentrations were based on ranges provided by the Mayo Clinic (https://www.mayocliniclabs.com/). Based on clinical advice, the upper range for 1,25(OH)_2_D was set at 80 pg/mL, in line with Mayo Clinic ranges.

**Supplemental Table 3. Baseline demographics and disease characteristics in patients with** **eGFR <90** **ml/min/1.73 m^2^ and eGFR ≥ 90** **ml/min/1.73 m^2^**

|  | Children | | | Adults | | |
| --- | --- | --- | --- | --- | --- | --- |
| ​ | **eGFR <** **90***  **N=27** | **eGFR** **≥ 90**  **N=166** | **Total**  **N=​193** | **eGFR < 90**  **N=42** | **eGFR ≥ 90**  **N=275** | **Total**  **N=​317** |
| **Age at XLH Diagnosis, years**  mean (SD)  median | n=26  1.5 (1.6)  1.1 | n=159  1.6 (2.0)  1.2 | n=185  1.6 (1.9)  1.2 | n=41  13.4 (20.0)  3.6 | n=228  8.7 (14.2)  2.0 | n=269  9.4 (15.3)  2.2 |
| **Age at baseline, years**  mean (SD)  median | 8.8 (4.8)  9.1 | 7.5 (3.8)  7.6 | 7.6 (4.0)  7.9 | 52.2 (13.5)  51.0 | 38.5 (12.1)  37.7 | 40.3 (13.1)  39.0 |
| **Female, n (%)** | 18 (67) | 91 (55) | 109 (57) | 31 (74) | 200 (73) | 231 (73) |
| **Race, n (%)** |  |  |  |  |  |  |
| White | 20 (74) | 128 (77) | 148 (77) | 36 (86) | 199 (72) | 235 (74) |
| Black | 2 (7) | 7 (4) | 9 (5) | 0 (0) | 5 (2) | 5 (2) |
| Asian | 0 (0) | 9 (5) | 9 (5) | 1 (2) | 38 (14) | 39 (12) |
| Other/ Unknown | 5 (19) | 22 (13) | 27 (14) | 5 (12) | 33 (12) | 38 (12) |
| **Region, n (%)** |  |  |  |  |  |  |
| *United States | 14 (52) | 97 (58) | 111 (58) | 30 (71) | 142 (52) | 172 (54) |
| Canada | 3 (11) | 25 (15) | 28 (15) | 5 (12) | 24 (9) | 29 (9) |
| Europe | 6 (22) | 16 (10) | 22 (11) | 2 (5) | 46 (17) | 48 (15) |
| Asia-Pacific | 1 (4) | 15 (9) | 16 (8) | 0 (0) | 35 (13) | 35 (11) |
| Latin America | 3 (11) | 13 (8) | 16 (8) | 5 (12) | 28 (10) | 33 (10) |
| **Height z-score**  mean (SD)  median | -1.9 (2.4)  -1.4 | -1.9 (1.2)  -1.9 | -1.9 (1.4)  -1.9 | n=40  -1.9 (1.5)  -1.6 | n=270  -2.4 (1.7)  -2.4 | n=310  -2.3 (1.7)  -2.3 |

The number of patients in each group is listed if there were any missing data. *Includes 2 infants with eGFR <90, which was within normal levels for this age range.

**Supplemental Table 4. Baseline biochemical values in patients with eGFR <90 ml/min/1.73 m^2^ and eGFR ≥ 90** **ml/min/1.73 m^2^**

|  | **Children** | | | **Adults** | | |
| --- | --- | --- | --- | --- | --- | --- |
|  | **eGFR < 90*** | **eGFR ≥ 90** | **Total** | **eGFR < 90** | **eGFR ≥ 90** | **Total** |
|  | **n=27** | **n=166** | **n=193** | **n=42** | **n=275** | **n=317** |
| **Serum phosphorus** | n=27  23 (85)  2.6 (0.7)  2.5 | n=166  163 (98)  2.5 (0.4)  2.5 | n=193  186 (96)  2.5 (0.5)  2.5 | n=42  27 (64)  2.4 (0.6)  2.3 | n=275  237 (86)  2.1 (0.4)  2.1 | n=317  264 (83)  2.1 (0.4)  2.1 |
| Patients below LLN, n (%)  mg/dL, mean (SD)  median |  |  |  |  |  |  |
| **TmP/GFR** | n=13  13 (48)  1.9 (0.3)  1.8 | n=92  90 (54)  2.1 (0.5)  2.1 | n=105  103 (53)  2.1 (0.4)  2.0 | n=11  10 (24)  1.6 (0.6)  1.4 | n=147  142 (52)  1.7 (0.4)  1.7 | n=158  152 (48)  1.7 (0.4)  1.7 |
| Patients below LLN, n (%) |  |  |  |  |  |  |
| mg/dL, mean (SD)  median |  |  |  |  |  |  |
| **Serum calcium** | n=19  4 (15)  10.0 (0.5)  9.9 | n=125  7 (4)  9.6 (0.4)  9.6 | n=144  11 (6)  9.7 (0.5)  9.7 | n=23  1 (2)  9.5 (0.6)  9.4 | n=176  2 (1)  9.2 (0.5)  9.1 | n=199  3 (1)  9.2 (0.5)  9.1 |
| Patients above ULN, n (%) |  |  |  |  |  |  |
| mg/dL, mean (SD)  median |  |  |  |  |  |  |
| **Serum iPTH** | n=26  4 (15)  55.5 (52.1)  43.5 | n=163  32 (19)  49.2 (29.2)  42.4 | n=189  36 (19)  50.0 (33.2)  42.5 | n=42  25 (60)  103.4 (76.5)  85.2 | n=273  152 (55)  86.5 (57.3)  77.1 | n=315  177 (56)  88.8 (60.3)  78.0 |
| Patients above ULN, n (%) |  |  |  |  |  |  |
| pg/mL, mean (SD)  median |  |  |  |  |  |  |
| **ALP** | n=27  21 (78)  435 (175)  400 | n=163  133 (80)  469 (156)  466 | n=190  154 (80)  464 (159)  465 | n=23  12 (29)  114 (42)  108 | n=181  86 (31)  127 (72)  110 | n=204  98 (31)  126 (70)  110 |
| Patients above ULN, n (%)  U/L, mean (SD)  median |  |  |  |  |  |  |
| **1,25(OH)_2_D** | n=23  7 (26)  42.2 (26.3)  33.6 | n=158  28 (17)  44.5 (17.6)  41.9 | n=181  35 (18)  44.2 (18.8)  41.4 | n=39  12 (29)  39.8 (22.2)  36.6 | n=257  56 (20)  38.9 (15.9)  37.6 | n=296  68 (22)  39.0 (16.8)  37.3 |
| Patients below LLN |  |  |  |  |  |  |
| pg/mL, mean (SD)  median |  |  |  |  |  |  |
| **25-hydroxyvitamin D** | n=15 | n=109 | n=124 | n=11 | n=147 | n=158 |
| Patients < 30 ng/mL, n (%) | 6 (22) | 47 (28) | 53 (28) | 4 (10) | 104 (38) | 108 (34) |
| Patients < 20 ng/mL, n (%) | 0 (0) | 11 (7) | 11 (6) | 2 (5) | 50 (18) | 52 (16) |
| ng/mL, mean (SD)  median | 35.9 (13.8)  34.8 | 30.9 (9.1)  31.1 | 31.5 (9.8)  31.3 | 29.6 (9.4)  31.0 | 24.8 (10.8)  24.0 | 25.1 (10.8)  24.1 |

NC: nephrocalcinosis. Nephrocalcinosis was defined as a renal ultrasound score ≥1. LLN: lower limit of normal. ULN: upper limit of normal. TmP/GFR: tubular maximum for phosphate reabsorption per GFR. iPTH: intact parathyroid hormone. ALP: alkaline phosphatase. Normal ranges are shown in Supplemental Table 1. *Includes 2 infants with eGFR <90, which was within normal levels for this age range.

**Supplemental Figure**

**Supplemental Figure 1.** Sensitivity analysis of eGFR in children and adults when those with eGFR >190 ml/min/1.73 m^2^ are excluded. Circles within the shaded bars indicate means. *P<0.001. Includes 2 infants with eGFR <90 ml/min/1.73 m2, which was within normal levels for this age range.


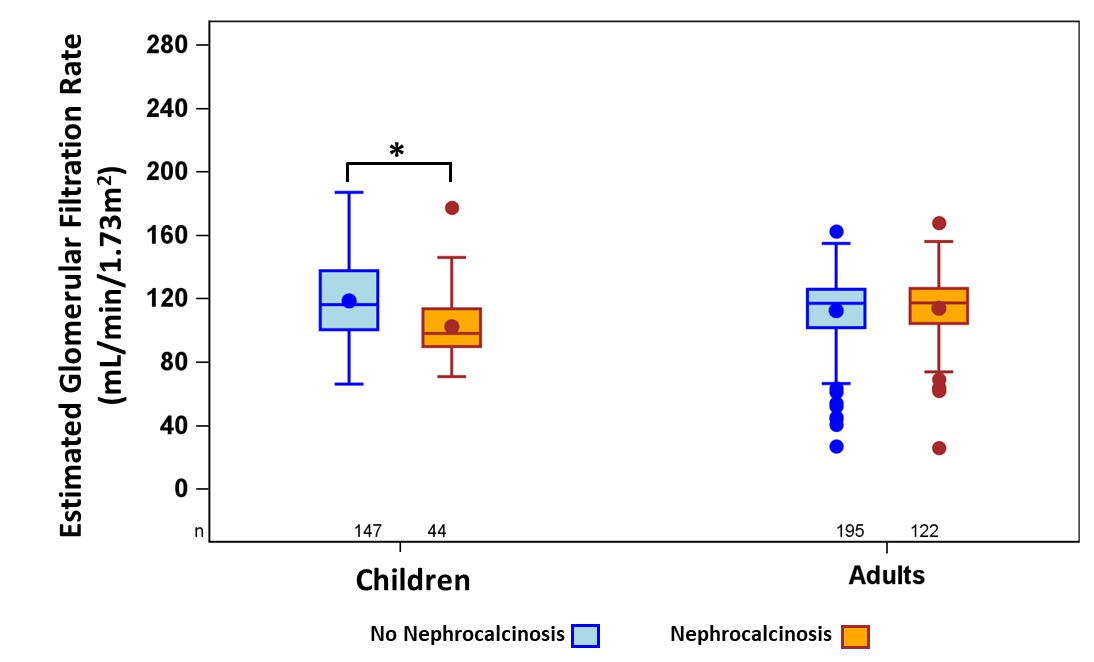

Supplement: Supplemental_Information_zjae127 [file supplemental_information_zjae127.docx]
